# Development and Validation of the Nursing Students’ Rights Awareness Scale in Clinical Practice: A Scale Development Study

**DOI:** 10.3390/healthcare9101323

**Published:** 2021-10-04

**Authors:** Sung-Hee Park, Mi-Young Choi

**Affiliations:** 1 Department of Nursing, Kunsan National University, Gunsan 54150, Korea; shpark@kunsan.ac.kr; 2 Department of Nursing Science, Chungbuk National University, Cheongju 28644, Korea

**Keywords:** human rights, nursing, practical, students, nursing, validation study

## Abstract

Nursing students perform clinical training in a variety of clinical practice fields, so their rights are important. Efforts are needed to accurately identify and measure the awareness of nursing students’ rights. This study aimed to develop and evaluate the psychometric properties of nursing students’ rights awareness scale. The study procedure was carried out in four stages: the concept analysis, item development, scale development, and scale evaluation stage. First, in the concept analysis stage, the attributes of the concept were derived. Second, in the item development stage, preliminary items were derived, and the content validity was verified. Third, in the scale development stage, a preliminary and main survey were conducted, and item reduction was implemented. Fourth, in the scale evaluation stage, construct validity and reliability were verified. The collected data were analyzed using the SPSS 24.0, and item analysis was conducted using skewness, kurtosis, and item-total correlation scores. The construct validity was assessed by exploratory factor analysis, convergent validity, and divergent validity. The reliability was analyzed using Cronbach’s alpha coefficient and composite reliability. The final version of the scale was tested on 240 nursing students from three cities in South Korea. From the construct validity, three factors and 14 items were selected. The factors included “the rights to be protected, cared for, supported, and respected” (eight items), “the rights to be recognized as a member of a nursing team” (three items), and “the rights to learn” (three questions). The reliability of the scale was also verified. Through this study, the scale was developed to identify the rights of nursing students in clinical practice. The implication of this study is that it has laid the foundation for measuring the rights of nursing students applicable in clinical settings for the first time. The limitation of this study is that data were collected in some regions. Therefore, it is necessary to verify the validity and reliability of the scale in various cultures.

## 1. Introduction

Nursing students’ rights in clinical practice refer to rights to learn, and be safe and protected from infection and accident, and have a support educational system in the clinical field [1,2].

Clinical training is an essential element of nursing education because it enables nursing students to apply theories in clinical practice [3,4]. Receiving clinical training in a supportive environment is the foundation to become a good nurse [5,6].

However, nursing students are often exposed to human rights violations, such as neglect, discrimination, and harassment during clinical training [7,8,9]. As a result, nursing students are experiencing a violation of their rights to learn that prevents them from receiving supportive education [6,8].

In addition, it is said that even if there is a case where they are exposed to safety and infection without protection, they cannot assert their rights due to a lack of awareness. Therefore, to ensure a safe and supportive clinical environment, efforts are needed [2,10]. For this, an appropriate scale is needed to analyze and measure the concept of awareness of the rights of nursing students.

Although previous studies were reviewed, many studies qualitatively analyzed the experiences of clinical practice [5,7,11]. Importantly, there was no scale to analyze and measure the concept of rights of nursing students. As a result, various studies were not attempted, and the level of awareness of their rights was insufficient [2,10].

Therefore, it will be necessary to make efforts to increase the rights of nursing students, and as a basic step, research on the scale development is required [2]. To analyze related concepts, it is necessary to proceed with a theoretical phase through literature reviews and a fieldwork phase through in-depth interviews using a hybrid model [12,13]. 

This study used a hybrid model developed by Schwartz-Barcott and Kim to examine the concept and its attributes. The hybrid model is an appropriate method for concept analysis and consists of three phases. Phase 1 is theoretical, and after analyzing the literatures, attributes of the concept are derived. Phase 2 is the fieldwork, and in-depth interviews are conducted. In the last phase, which is the final analysis, the attributes derived from phases 1 and 2 are compared and analyzed to finally derive the attributes of the concept [12]. This study analyzed the concept using a hybrid model and used it to develop the measurement scale.

The scale of this study was developed based on the hybrid model, psychometric theory, and previous scale development research. According to them, this study was carried out in four stages: the concept analysis, item development, scale development, and scale evaluation stage. First, in the concept analysis stage, the attributes of the concept were derived. Second, in the item development stage, preliminary items were derived, and the content validity of the preliminary items was verified by experts. Third, in the scale development stage, a preliminary survey was conducted on 10 nursing students. Then, the main survey with 240 nursing students was conducted and item reduction was implemented through item-total correlation, skewness, and kurtosis. Fourth, in the scale evaluation stage, construct validity and reliability were verified [12,14,15]. 

This study aimed to develop and evaluate the psychometric properties of nursing students’ rights awareness scale based on the results of concept analysis. The research question of this study is “What are the characteristics of awareness of the nursing student’s right?”. 

The structure of this paper is as follows. In Section 2, we present the research methods including concept analysis, item development, scale development, and scale evaluation stage. Section 3 presents the research results, Section 4 presents the discussion, limitations, and implications. Section 5 presents the conclusions. 

## 2. Materials and Methods

### 2.1. Research Approach

This study used a mixed-method research methodology. The reason is that concept analysis using a hybrid model and questionnaire survey for scale development were conducted together. Specifically, in-depth interviews with subjects were conducted in the fieldwork phase of the hybrid model, so a qualitative methodology was used, and a quantitative methodology was used in the questionnaire survey for scale development.

### 2.2. Study Procedure

**Figure 1:**
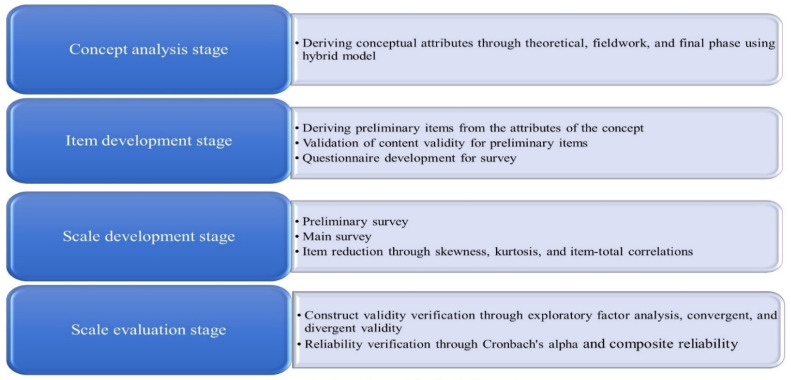
Overview of the study procedure.

The study procedure of this study is shown in Figure 1.

#### 2.2.1. Concept Analysis Stage

Through a concept analysis using a hybrid model [12], the attributes of awareness of nursing students’ rights in clinical practice were confirmed. First, the five attributes were derived in the theoretical phase: (i) the rights to learn, (ii) the rights to be protected from infections and safety accidents, (iii) the rights to be cared for and supported, (iv) the rights to be respected, and (v) the rights to be recognized as a member of a nursing team. Second, 10 nursing students were interviewed at the fieldwork phase, and the attributes derived from the theoretical phase were reconfirmed. Third, in the final phase, the five attributes were confirmed by synthesizing the previous two phases.

#### 2.2.2. Item Development Stage

A total of 43 preliminary items were created based on the five attributes derived from the concept analysis stage. There were 10 items on the “rights to learn,” five items on the “rights to be protected from infections and safety accidents”, 12 items on the “rights to be cared for and supported,” nine items on the “rights to be respected,” and seven items on the ”rights to be recognized as a member of a nursing team”.

The content validity of the preliminary items was examined by five experts (professors of nursing departments with more than 20 years of clinical and educational experience). Content validity was verified using the item content validity index and sum content validity index. Only items with a 1.0 content validity index were selected as final items [14,15,16]; thus, 26 items were selected. Seven items were from the rights to learn, five from the rights to be protected from infections and safety accidents, six from the rights to be cared for and supported, five from the rights to be respected, and three items from the rights to be recognized as a member of a nursing team.

##### Questionnaire Development

The questionnaire in this study consisted of demographic characteristics (9 items) and items measuring nursing students’ rights awareness in clinical practice (5 factors, 43 items). The demographic characteristics question was based on related previous studies [2,10], and the questions related to nursing students’ rights awareness were made based on the five attributes derived from the concept analysis and 43 main statements of in-depth interview participants. 

In-depth interviews questions were as follows. “What do you think nursing students’ rights in clinical practice are?”, “What are some rights you have experienced as a nursing student in clinical practice?”, and “Have your rights as a nursing student in clinical practice been violated?”. Interview questions were drafted by the authors based on a previous study [2] and confirmed by two nursing professors.

Before conducting the main survey, a preliminary survey was first conducted on 10 nursing students to check their understanding of the questionnaire. For items with low understanding, the sentences were modified with easier terms.

#### 2.2.3. Scale Development Stage

For the preliminary survey, convenient sampling was performed with 10 nursing students to confirm their understanding of the selected preliminary items. For the items that they indicated having low understanding, the sentences were modified before the completion of the final questionnaire survey. The main survey was conducted through convenience sampling of 240 nursing students enrolled in the nursing department.

Skewness, kurtosis, and item-total correlations were analyzed for item reduction. In this analysis, items with an absolute value of three or more for skewness and an absolute value of seven or more for kurtosis were excluded. The item-total correlations included items with a value of 0.30 or higher [14,15,17].

#### 2.2.4. Scale Evaluation Stage

Scale evaluation was verified by construct validity and reliability [14,15]. The construct validity was confirmed by exploratory factor analysis with the maximum likelihood method and varimax rotation. The number of participants required to conduct exploratory factor analysis was 200 or more [18]. In this study, 240 subjects were used. Items with a commonality of less than 0.50 were removed, and only items with eigenvalues of 1.00 or higher were extracted as factors. In addition, only items with a factor loading of 0.50 or more and cross factor loading 0.50 or less were included as scale items. The AVE (average variance extracted) was checked for convergent validity. The cross factor loading and Fornell and Larcker criterion were checked for divergent validity. Convergent validity is verified when the AVE value is greater than 0.50. In the Fornell and Larcker criterion, divergent validity is secured when the AVE is greater than the square of correlation of factors [19,20,21,22]. 

To test reliability, Cronbach’s alpha for the scale and subfactors were checked. [14,15]. In addition, the composite reliability was also confirmed. All reliability values should be greater than or equal to 0.70 to ensure the consistency of scale [20,21,22].

### 2.3. Study Participants

The participants of this study were 240 nursing students enrolled in the fourth year at the department of nursing, and the criteria for selecting the participants were as follows: The participants had to have the experience of at least two semesters (1 year) of clinical training with expressed willingness to participate in the research. Selecting nursing students with experience of at least two semesters as inclusion criteria was based on the results of previous studies [6,23].

In this study, a survey was conducted among 240 nursing students considering the dropout rate, of which 222 surveys were used for analysis, excluding 18 surveys with insufficient data.

### 2.4. Data Collection

#### 2.4.1. In-Depth Interviews

Data were collected from 15 July to 20 August 2020. Participants were recruited with the help of an administrative assistant after obtaining permission from the head of the department. Participants were recruited using the department’s online chat system with the help of an administrative assistant. 

Face-to-face in-depth interviews were conducted after participants signed their consent forms. The purpose and procedure of this study, its anonymity and confidentiality, and the possibility of withdrawing from the study were explained before the interviews. 

The interviews were conducted at a location agreed to by each participant, such as a school lounge room and café, where the participants could talk comfortably. Each interview lasted 40–60 min. 

#### 2.4.2. Main Survey

From 10 December 2020 to 15 February 2021, the data were collected through a survey of fourth-year nursing students in nursing departments in Jeonbuk, Seoul, and Gyeonggi-do province. 

For the survey, the authors visited the school and conducted a face-to-face survey. Participants were recruited with the help of administrative assistants after obtaining permission from the heads of the department. Participants were recruited using the departments’ online chat system with the help of administrative assistants. 

The time required to complete the questionnaire was 10–15 min. The data were collected after obtaining participants’ consent. The purpose and procedure of this study, its anonymity and confidentiality, and the possibility of withdrawing from the study were explained before the survey.

### 2.5. Data Analysis

The collected data were analyzed using the SPSS 24, and the participants’ characteristics were analyzed using descriptive statistics. Skewness, kurtosis, and item-total correlations were analyzed. The items with an absolute value of three or more for skewness and an absolute value of seven or more for kurtosis were excluded. The item-total correlations included items with a value of 0.30 or higher.

The Kaiser–Meyer–Olkin (KMO) and Bartlett’s sphericity tests were performed to confirm whether the collected data were suitable for exploratory factor analysis. An exploratory factor analysis was performed to test the construct validity, and the maximum likelihood method and varimax rotation were used. Items with a commonality of less than 0.50 were removed, and only items with eigenvalues of 1.00 or higher were extracted. In addition, only items with a factor loading of 0.50 or more and cross factor loading of 0.50 or less were included. The factor loading value must be greater than the cross factor loading value.

In addition, the construct validity was confirmed by convergent and divergent validity. The AVE was checked for convergent validity. The Fornell and Larcker criterion was checked for divergent validity. Convergent validity is verified when the AVE value is greater than 0.50. Divergent validity is secured when the AVE is greater than the square of the correlation of factors.

To test reliability, Cronbach’s alpha for the scale was checked. The composite reliability was also examined. All values should be greater than or equal to 0.70.

### 2.6. Ethical Considerations

Approval was obtained from the Bioethics Committee of the researcher’s institution prior to the start of the study (1040117-2-2—6-HR-009-01).

## 3. Results

### 3.1. Characteristics of the Participants

Among the participants of this study, 94.6% were women and 5.4% were men. The average age was 23.76 ± 3.52 years. The duration of the clinical training experience was distributed between 22 and 26 weeks, and the degree of satisfaction with clinical training was moderate (49.5%) and satisfactory (35.6%) Most of the previous clinical training experience was more than one year (84.7%), and the participation period was mostly spring and fall semesters (Table 1).

### 3.2. Item Analysis

By examining the absolute values of the skewness and kurtosis of the 26 items, the kurtosis of item numbers 8 (7.70) and 9 (9.51) was found to be 7 or higher; thus, these items were removed. The correlation between the item and total score of all items was 0.48–0.78, with no item having a value less than 0.30 value. Therefore, 24 items were used for exploratory factor analysis.

### 3.3. Test of Construct Validity

Tests for Kaiser–Meyer–Olkin (KMO) and Bartlett’s sphericity tests were performed. The data were found to be suitable for factor analysis, as KMO was 0.93 and Bartlett’s sphericity verification was *p* < 0.05. Based on the communality finding of all items, six items were removed, which included items 1 (communality 0.33), 5 (communality 0.45), 12 (communality 0.41), 17 (communality 0.48), 21 (communality 0.40), and 24 (communality 0.41). The second exploratory factor analysis was conducted using the remaining 18 items.

As a result, item numbers 14 and 26 were loaded into two factors at the same time, so the items were deleted, and a 3rd-factor analysis was performed with 16 items. Item numbers 6 and 7 were also loaded into two factors, two items were deleted.

After that, a fourth-factor analysis was performed with a total of 14 items. As a result, the factor loading of all items was more than 0.50, and the cross-factor loading was less than 0.50. There were no more deleted items, and a total of 14 items were confirmed as final scale items. Three factors with an eigenvalue of 1.00 or higher were extracted, and the total explained variance was 63.3% (Table 2).

Factor 1 had eight items, which included information on infected patients, guidelines for responding to incidents and accidents, solutions to students’ requests, establishment of student advocacy system, support in clinical training for students, the rights to be free from violence, and be treated respectfully. Therefore, it was titled “The rights to be protected, cared, supported, and respected”.

Factor 2 included three items: the rights to ask questions to the nurse during clinical training and hear the answers, learn according to the standardized clinical training manual, and receive a systematic orientation from a unit manager at the beginning of the clinical training. Therefore, it was titled “the rights to learn”.

Factor 3 included three items, including the rights to not perform tasks that are not directly relevant to the clinical training, be addressed by a title that shows respect, and be guaranteed of the mealtime. Therefore, it was titled “the rights to be recognized as a member of a nursing team”.

In addition, the construct validity was confirmed by convergent and divergent validity. The AVE was checked for convergent validity. The Fornell and Larcker criterion was checked for divergent validity. Convergent validity was verified as all AVE values were above 0.50. Divergent validity was secured when the AVE was greater than the square of correlation of factors (Table 3).

### 3.4. Test of Reliability

The Cronbach’s alpha of the 14 items was found to be 0.92. A suitable level of reliability was evident as the reliability for factor 1 was 0.92, factor 2 was 0.83, and factor 3 was 0.82. The composite reliability was also examined, and all values were above 0.70 (Table 4).

## 4. Discussion

This study was conducted to develop a scale to measure nursing students’ awareness of their rights in clinical practice and to examine the validity and reliability of the scale. A scale with 14 items across the three factors was derived. The three factors are as follows: “the rights to be protected, cared for, supported, and respected,” “the rights to learn“, “the rights to be recognized as a member of a nursing team”.

First, factor 1 was “the rights to be protected, cared for, supported, and respected”. It contains 8 items. Specifically, three items measured the rights to receive attention, support, and advocacy during clinical practice, two items were the rights to be protected from violence and infection. Two items were guidelines and reporting related to accidents, and one item was the rights to practice with respect.

Prior studies confirmed that nursing students needed the attention and support of nurses and unit managers, and they want clinical instructors and schools to protect them from risk [2,3,24] and to guarantee their rights in clinical practice [25].

In addition, they wanted to practice in a safe environment without infection and violence [2,24,25,26] and hoped that there would be an accident-related advocacy system [2]. In previous studies that qualitatively analyzed the practical experience of nursing students, they wanted to practice while being respected without being discriminated or ignored [3,8,11,27].

However, nursing students were often exposed to violence, such as being yelled at by nurses or experiencing discrimination [8,27,28]. Nursing students were also worried about being infected, as the hospitals did not provide information regarding patients [24].

Nursing students want to practice in a safe environment with support and protection, but they often experience unprotected when exposed to accidents or violence. Furthermore, there are students who practice while being discriminated and ignored [2,8,27]. This seems to be due to the lack of awareness of nursing students’ rights in clinical practice. It is necessary to pay attention to the rights of nursing students. By applying the scale developed through this study in nursing education and practical fields, it is necessary to lay the foundation for the promotion and improvement of awareness of the rights of nursing students.

Second, factor 2 was “the rights to learn”. It contains three items and consisted of the rights to ask questions to the nurse, to learn according to the standardized clinical manual, and to receive a systematic orientation from a unit manager.

Nursing students have a desire to learn through clinical practice [29]. For effective practice, nurses and unit managers are expected to actively participate in education and to educate them [2,5,30]. In previous studies, nursing students were perceived to need academic support from unit managers and nurses [30,31]. Specifically, they wanted to be encouraged to try out nursing skills and to answer questions well. In addition, standardized manuals and orientations were desired.

However, nursing students reported that nurses were uninterested in clinical education. Furthermore, they perceived that the opportunities for learning in practice were very insufficient. [2,8,24]. Clinical training is an essential element of nursing because it enables nursing students to apply theories in clinical practice [3,4]. Therefore, nursing educators will have to strive to increase nursing skills opportunities, and long-term plans should be established for systematic and standardized practical education.

Third, factor 3 was “the rights to be recognized as a member of the nursing team”. It contains three items including the rights to not perform tasks that are not relevant to the training, to be called by a title of respect, and to be guaranteed of mealtimes.

In the concept analysis procedure of this study, nursing students said that it is inconvenient to do tasks that are not related to practice such as patient’ private errands. They also said that calling ’student nurse’ makes them feel like a nursing team. In addition, because they assist with nursing work, it is necessary to guarantee mealtimes including rest.

Previous studies have also stated that mealtime and break time should be guaranteed, and private errands of patients should not be run [2,5]. In a study conducted to confirm nursing students’ expectations for effective clinical education, students perceived those nurses delegated their duties or introduced students to staff or patients as effective education [11]. What was newly derived from this study was that it was necessary to call them by a respectful title. It is said that when they are called ’student’ or even ’hey’, they feel ignored. Nursing students recognized that it was necessary to be called a ’student nurse’ because they delegated and performed the duties of a nurse.

Therefore, it is necessary to establish a system that accepts nursing students as members of the nursing team and delegates and assigns nursing tasks within a trusting relationship.

The limitation of this study is that the process of retesting the construct validity through a confirmatory factor was not performed. Therefore, it is necessary to supplement this limitation through large-scale studies. Moreover, the limitation of this study is that data were collected in some regions. Therefore, it is necessary to verify the validity and reliability of the scale in various cultures in the future.

The academic implications of this study are as follows. First, it was an opportunity to realize the need to increase awareness of the rights of nursing students in clinical practice. Nursing students want to be more protected and supported in the clinical setting. They also want to learn while being respected. Therefore, it is necessary for schools to find ways to continuously enhance the rights of nursing students. Second, it was realized that schools need to have a system that guarantees the rights of nursing students in clinical education. In clinical practice, nursing students are often exposed to infections, incidents, and situations of violence, but there were cases where the system to manage them was insufficient. Therefore, efforts to supplement and improve these problems will be continuously required.

The practical implications of this study are as follows. First, it was that it developed the scale to measure nursing students’ rights in clinical practice and laid the foundation for its application in various clinical settings. It is necessary to provide basic data for developing a program to improve the rights of nursing students through various research attempts to confirm the relationship between the rights of nursing students and other variables. Second, this study suggests that hospitals should make efforts so that nursing students can practice and learn in a safe environment. For this, it is necessary to pay attention to securing the nurse system in charge of student education and the educational expertise of the unit manager.

## 5. Conclusions

This study was conducted to develop a scale to measure nursing students’ awareness of their rights in clinical practice. As a result, a scale consisting of three factors and 14 items was derived. The factors included “the rights to be protected, cared for, supported, and respected” (eight items), “the rights to learn” (three items)”, and “the rights to be recognized as a member of a nursing team” (three items).

The conclusions of this study are as follows: First, nursing students have the right to practice while being respected in a protective and supportive environment. To ensure this, schools and hospitals will have to work together to maintain a safe practice environment. Second, nursing students have the right to learn to apply theory to practice. Specifically, they have the right to promote opportunities for hands-on nursing skills and receive education through systematic and standardized practice manuals. To ensure this, schools and hospitals should prepare standardized practice manuals and come up with ways to expand opportunities for participation in nursing skills. Third, nursing students have the right to practice as a member of a nursing team. Hospitals need to have a system that can formally introduce nursing students to wards and patients and delegate nursing assistant tasks in a trusting relationship. In addition, it should be ensured that respectful titles are used, and rest and mealtimes are guaranteed.

The scale developed in this study is suitable for measuring the nursing students’ rights in clinical practice. This scale may be used in various nursing education and clinical fields in the future. This study was validated for Korean nursing students. This will allow cross-cultural studies of rights awareness in nursing students.

## Figures and Tables

**Table 1:** Characteristics of the participants (*n* = 222).

Characteristics	*n* (%)	M ± SD
Gender		
Female	210 (94.6)	
Male	12 (5.4)	
Age (years)		
20–29	210 (94.6)	23.76 ±3.52
>30	12 (5.4)	
Clinical training experience		
22 weeks	32 (14.4)	24.79 ±1.46
24 weeks	70 (31.5)	
26 weeks	120 (54.1)	
Satisfaction with nursing		
Satisfied	135 (60.8)	
Moderate	77 (34.7)	
Dissatisfied	10 (4.5)	
Satisfaction with clinical training		
Satisfied	79 (35.6)	
Moderate	110 (49.5)	
Dissatisfied	33 (14.9)	
School location		
Jeonbuk	34 (15.3)	
Seoul	151 (68.0)	
Gyeonggi-do	37 (16.7)	
Previous clinical training		
1 year	34 (15.3)	
Over 1 year	188 (84.7)	
Participation period of clinical training		
Spring and fall semester	188 (84.7)	
Summer and winter semester	34 (15.3)	
Completion of rights-related training		
Yes	144 (64.9)	
No	78 (35.1)	

**Table 2:** Exploratory factor analysis (*n* = 222).

(Item Number) Item	Factor Loading		
	Factor 1	Factor 2	Factor 3
(11) I have the rights to receive information in advance on the response instructions in case of an incident or an accident.	0.74	0.36	0.20
(20) I have the rights to be treated respectfully.	0.72	0.21	0.29
(16) Universities must have a system implemented for students to report any disadvantages or injustices experienced immediately.	0.67	0.28	0.30
(13) The clinical instructor must pay attention to solving the students’ suggestions or requests.	0.65	0.19	0.40
(15) The university must have a system to respond systematically to the clinical training institution by representing the students in case of incidents and accidents that occur during the clinical training period, and to advocate for the students.	0.65	0.34	0.21
(10) I have the rights to receive information about infection status of patients in advance.	0.65	0.42	0.16
(19) I have the right to be free from verbal, physical, and sexual assault.	0.65	0.24	0.25
(18) The clinical instructor must provide attention and support in guiding the students during clinical training.	0.55	0.32	0.45
(2) I have the rights to ask questions to the nurse during clinical training and hear the answers.	0.20	0.77	0.16
(3) I have the rights to learn according to the standardized clinical training manual.	0.24	0.72	0.16
(4) I have the rights to receive a systematic orientation from a unit manager at the beginning of the clinical training.	0.38	0.70	0.09
(23) I have the rights to be addressed by a title that shows respect to the students (e.g., a student nurse).	0.23	0.14	0.88
(22) I have the rights to not perform tasks that are not directly relevant to the clinical training.	0.20	0.16	0.73
(25) I have the rights to have the mealtime guaranteed.	0.34	0.13	0.61
Eigen value	7.31	1.52	1.06
Explained variance (%)	29.6	17.3	16.4
Total explained variance (%)	29.6	46.9	63.3

**Table 3:** Convergent and divergent validity.

Factors	Factor 1 r^2^ (*p*)	Factor 2 r^2^ (*p*)	Factor r^2^ (*p*)	AVE\
Factor 1	1			0.77
Factor 2	0.50 (<0.01)	1		0.86
Factor 3	0.41 (<0.01)	0.18 (<0.01)	1	0.86

**Table 4:** Reliability (*n* = 222).

Subfactors	Item Number	Cronbach’s Alpha	Composite Reliability
Factor 1: The rights to be protected, cared, supported, and respected	11 20 16 13 15 10 19 18	0.92	0.96
Factor 2: The rights to learn	2 3 4	0.83	0.95
Factor 3: The rights to be recognized as a member of a nursing team	23 22 25	0.82	0.95
Total		0.92	

## Data Availability

The data presented in this study are available on request from the corresponding author. The data are not publicly available due to ethical reason.
